# Dr. George F. Carmichael on the Treatment of Obstinate Constipation, with Cases

**Published:** 1842-05-07

**Authors:** George F. Carmichael


### *On the Treatment of Obstinate Constipation.*

By George F. Carmichael, M. D.

To the Editors of the Medical Examiner.

Gentlemen,—I forward the results of practice in one or two cases which have came under my care. If they are of any practical importance, and are in your estimation worthy of insertion in your valuable journal, you are at liberty to publish them.

Most respectfully, your obt. servt.,

Geo. F. Carmichael.

*Fredericksburg*, *Va.*, *April 27th*, 1842.

—

Captain C., a seafaring man, has had permanent stricture of the rectum for five or six years, to such a degree as to subject him to habitual difficulty in evacuating his bowels, and occasionally to complete obstruction and retention of the fæces. At the time I was called to see him he had had no evacuation from his bowels for five days, and at that time was suffering greatly from aggravated symptoms of constipation, viz., violent pain and enormous distension of the abdomen, sick stomach, constant vomiting, great thirst, sleeplessness, jactitation, inability to remain in a recumbent posture, oppressed and anxious countenance, &c. Previously to my visit he had taken numerous doses of purgative medicine, some of which were retained, and others rejected.

*Prescription.*—Bloodletting to ℥xx; warm bath; purgative draughts; enemata. No relief being induced by these means, and the symptoms increasing, I introduced the gum elastic stomach tube of a patent injecting apparatus, its whole length, (twenty-four inches, per anum,) into the bowels, and threw up gently and slowly about a quart of warm soap and water. This was retained for some minutes, and its reflux then permitted through the tube. Whenever the eyelets of the tube became obstructed by undissolved fæcal matter, they were cleared by injecting a little warm water through it. In this manner the accumulated fæces, (the cause of the symptoms,) dissolved in the soap and water, were removed in a few minutes, and the patient expressed himself entirely relieved and immediately sunk to sleep. No farther treatment was necessary.

In a second case the same proceeding procured immediate relief, after the patient had been subjected for a whole week,—he being in the greatest distress,—to every variety and mode of treatment which is usually followed in such attacks,—and when he was almost given up in despair. In the second case there was no stricture.

Cases of the above character are commonly regarded by practitioners of medicine and friends of patients with peculiar anxiety and solicitude; and as I feel assured that a most frequent cause of such cases is accumulation of hard fæcal matter in the large intestines, particularly in the ascending colon, insoluble by the secretions of the lining membrane of the bowels—incapable of being urged along the intestinal tube, either from its bulk or loss of peristaltic power from over distension—and inaccessible to common lavements—I can recommend the above mode of injection for solution and removal of fæcal obstructions of the bowels as satisfactory and safe, at least so far as my experience goes. I of course do not pretend to originality in the adoption of this plan, but merely testify to its sufficiency in time of need, when the usual means are defective.

